# Development of an inducer-free, virulence gene promoter-controlled, and fluorescent reporter-labeled CRISPR interference system in *Staphylococcus aureus*

**DOI:** 10.1128/spectrum.00602-24

**Published:** 2024-08-20

**Authors:** Roni Miah, Mona Johannessen, Morten Kjos, Christian S. Lentz

**Affiliations:** 1 Department of Medical Biology and Center for New Antibacterial Strategies (CANS), UT- The Arctic University of Norway, Tromsø, Norway; 2 Faculty of Chemistry, Biotechnology and Food Science, Norwegian University of Life Sciences, Ås, Norway; University of Guelph, Ontario, Canada

**Keywords:** inducer-free CRISPRi, *Staphylococcus aureus*, bacterial cell population, dCas9 toxicity

## Abstract

The dCas9-based Clustered Regularly Interspaced Short Palindromic Repeats (CRISPR) interference (CRISPRi) gene regulation technique requires two components: a catalytically inactive Cas9 protein (dCas9) and a single-guide RNA that targets the gene of interest. This system is commonly activated by expressing dCas9 through an inducible gene promoter, but these inducers may affect cellular physiology, and accessibility and permeability of the inducer are limited in relevant model systems. Here, we have developed an alternative approach for CRISPRi activation in the clinical isolate *Staphylococcus aureus* USA300 LAC, where dCas9 was expressed through endogenous virulence gene promoters (vgp); coagulase, autolysin, or fibronectin-binding protein A. Additionally, we integrated a fluorescent reporter gene into the vgp-CRISPRi system to monitor the activity of the *dcas9*-controlling promoter. Testing the efficacy of vgp-CRISPRi by inducing growth arrest (when targeting penicillin-binding protein 1), downregulating target gene expression, or blocking coagulase-dependent coagulation of blood plasma, we provide a proof-of-concept demonstration that the virulence gene promoter-driven CRISPRi system is functional in *S. aureus*.

**IMPORTANCE:**

The presented inducer-free, endogenous virulence gene promoter-induced, dCas9-based Clustered Regularly Interspaced Short Palindromic Repeats (CRISPR) interference (CRISPRi system addresses several shortcomings related to the use of inducer-dependent systems such as effects on cell physiology or limitations in permeability, and it avoids the high, putatively toxic levels of dCas9 in CRISPRi systems controlled by strong, constitutive promoters.

## INTRODUCTION

CRISPR (Clustered Regularly Interspaced Short Palindromic Repeats) interference (CRISPRi) is an efficient and versatile tool for selectively perturbing gene expression on a genome-wide scale and thus dissecting their role in bacterial physiology or pathogenesis (1). The applications of CRISPRi are getting increasingly advanced, e.g., allowing for simultaneous regulation of expression of multiple genes (1, 2) construction of genetic oscillators (3, 4) quantification of genome-wide fitness using CRISPRi-seq (5–8) and screening approaches that may identify novel antimicrobial targets, including those for synergistic drug combinations (9, 10).

In the previously published CRISPRi-system, dCas9 (more specifically *Streptococcus pyogenes* SpydCas9) expression is under control of promotors that are activated by exogenous inducers such as IPTG (isopropyl-β-D-thiogalactopyranoside) (P*_lac_*) (9, 11, 12) or tetracycline (P*_tet_*) or its derivatives anhydrotetracycline (1) and doxycycline (7). The leaky character of P*_lac_* (9) and P_tet_ (13) promoters promote background dCas9 expression in the non-induced condition. Moreover, limitations in tissue and cell permeability render IPTG-inducible systems less suitable for use in certain model systems including animals (14). Undesired side-effects have been shown from the use of tetracyclines (15) or derivatives such as doxycycline (16) in mammalian cells, where they affect mitochondrial function (15) or can induce changes in gene transcription, metabolism, and cell proliferation (16), thus prompting caution for the use of these inducers in host-pathogen interaction studies.

Inducer-free alternatives CRISPRi system were developed, where dCas9 expression was controlled through strong constitutive gene promoters (17, 18). However, since it is well known that dCas9 provokes toxicity in many bacteria (19–24), its overexpression from strong constitutive promoters may cause undesirable phenotypes in *Staphylococcus aureus* cells. We reasoned that an alternative method for controlling CRISPRi system would be the use of virulence gene promoters. In such constructs, the dCas9 expression is switched on when the gene promoters are activated. Of note, the activity of many gene promoters has been shown not only to be conditional but also heterogeneous across cells within isogenic *S. aureus* subpopulations (25–28). This means that if *dcas9* is controlled through an endogenous promoter with heterogeneous activity across a bacterial cell population, the activity of the CRISPRi system will be restricted to those cells where this promoter is active and enable a pathway for specific manipulation of cellular subpopulations.

Here, we report the development of a virulence gene promoter-induced CRISPRi system (vgp-CRISPRi) that is inducer-free and fluorescent reporter-labeled. In this system, dCas9 expression is controlled by the promoters of well-known *S. aureus* virulence genes autolysin (*atl*), fibronectin-binding protein A (*fnbA*), and coagulase (*coa*). In combination with single-guide RNAs (sgRNAs) target genes that are involved in diverse phenotypes, we present a proof-of-concept validation that the vgp-CRISPRi system is functional in the clinically relevant methicillin-resistant *S. aureus* (MRSA) strain USA300 LAC.

## MATERIALS AND METHODS

### Materials

Standard agarose, type LE for routine gel electrophoresis was purchased from BioNordika, Oslo, Norway. DNA Ladder, 1.0 Kb Plus (100 bp to 15,000 bp) was purchased from Invitrogen. Primers were obtained from Sigma Life Science (Oslo, Norway), and sequencing reactions were performed using BigDye Terminator V3.1 (Life Technologies) and analyzed at the Faculty of Health Sciences, UiT - The Arctic University of Norway, Tromsø, Norway. The Wizard Genomic DNA Purification Kit was purchased from Promega. E.Z.N.A. Plasmid DNA Mini Kit I (Q – spin) was purchased from Omega. Phusion High-Fidelity DNA Polymerase and DreamTaq Green PCR Master Mix were purchased from Thermo Scientific. All restriction enzymes were purchased from New England Biolabs and used according to the manufacturer’s instructions. MagExtractor-PCR and Gel Clean up were purchased from Toyobo (Osaka, Japan). Rabbit Plasma (for the detection of Staphylocoagulase) was purchased from Bio-Rad. Roswell Park Memorial Institute (RPMI) 1640 medium and heat-inactivated, sterile-filtered Fetal Bovine Serum (FBS), Dulbecco’s Phosphate-Buffered Saline, PBS (D8537), and DNase I were purchased from Sigma-Aldrich. RNAprotect Bacteria Reagent and RNeasy Mini Kit were purchased from QIAGEN (Oslo, Norway). High-Capacity cDNA Reverse Transcription Kit was purchased from Applied Biosystems. All other materials were purchased from an analytical-grade commercial source and used according to the manufacturer’s instructions.

### Bacterial strains, media, growth conditions, plasmids, and transformation

The bacterial strains and plasmids used in this study are listed in Table 1 (*S. aureus* strains), S1 (*Escherichia coli* strains), and Table 2 (plasmids). The media used were lysogeny broth (LB; 244620, BD Difco), LB agar (244520, BD Difco), Tryptic Soy Broth (211825, BD Difco), and Tryptic Soy Agar (TSA; 236950, *BD* Difco). RPMI-1640 was purchased from Sigma-Aldrich (R8758) and used after supplementation with 10% FBS (F7524, Sigma-Aldrich) and is designated as RPMI+.

**TABLE 1:** *S. aureus* strains used in this study^*a*^

*S. aureus*	Genotype or description	Source or reference
USA300 LAC	Wild-type, coagulase-positive, community-acquired MRSA clone from the USA300 lineage, isolated from Los Angeles County (LAC)	(29)
RN4220	Restriction deficient derivative of NCTC8325-4	(30)
Fluorescent reporter strains		
MR 9	USA 300 LAC carrying pCM29-P*atl*-GFP, cmᴿ	This study
MR 10	USA 300 LAC carrying pCM29-P*fnbA*-GFP, cmᴿ	This study
MR 11	USA 300 LAC carrying pCM29-P*coa* -GFP, cmᴿ	This study
CRISPRi strains		
MR 15	USA 300 LAC carrying pLOW-P*lac*- d*cas*9, pCG248-sgRNA(*pbp1*), eryᴿ, cmᴿ	This study
MR 16	USA 300 LAC carrying pLOW-P*lac*- d*cas*9, pCM29-P*sarA* P1-*gfp*-sgRNA(*pbp1*), eryᴿ, cmᴿ	This study
MR 17	USA 300 LAC carrying pLOW-P*lac*- d*cas*9, pVL2336-sgRNA(NTC),eryᴿ, cmᴿ	This study
MR 18	USA 300 LAC carrying pLOW-P*lac*- d*cas*9, pCM29-P*sarA* P1-*gfp*-sgRNA(NTC), eryᴿ, cmᴿ	This study
MR 27	USA 300 LAC carrying pLOW-P*atl*- d*cas*9, pCM29-P*atl-gfp*-sgRNA (NTC), eryᴿ, cmᴿ	This study
MR 28	USA 300 LAC carrying pLOW-P*atl*- d*cas*9, pCM29-P*atl-gfp*-sgRNA(*pbp1*), eryᴿ, cmᴿ	This study
MR 29	USA 300 LAC carrying pLOW-P*fnbA*- d*cas*9, pCM29-P*fnbA-gfp*-sgRNA (NTC), eryᴿ, cmᴿ	This study
MR 30	USA 300 LAC carrying pLOW-P*fnbA*- d*cas*9, pCM29-*PfnbA-gfp*-sgRNA(*pbp1*), eryᴿ, cmᴿ	This study
MR 31	USA 300 LAC carrying pLOW-P*coa*- d*cas*9, pCM29-P*coa -gfp*-sgRNA(NTC), eryᴿ, cmᴿ	This study
MR 32	USA 300 LAC carrying pLOW-P*coa*- d*cas*9, pCM29-P*coa-gfp*-sgRNA(*pbp1*), eryᴿ, cmᴿ	This study
MR 23	USA 300 LAC carrying pLOW-P*lac*- d*cas*9, pCM29-P*sarA* P1-*gfp*-sgRNA(*coa*), eryᴿ, cmᴿ	This study
MR 24	USA 300 LAC carrying pLOW-P*atl*- d*cas*9, pCM29-P*sarA* P1-*gfp*-sgRNA(*coa*), eryᴿ, cmᴿ	This study
MR 25	USA 300 LAC carrying pLOW-P*fnbA*- d*cas*9, pCM29-P*sarA* P1-*gfp*-sgRNA(*coa*), eryᴿ, cmᴿ	This study
MR 26	USA 300 LAC carrying pLOW- P*coa* - d*cas*9, pCM29-P*sarA* P1-*gfp*-sgRNA(*coa*), eryᴿ, cmᴿ	This study

^*a*^ cmᴿ, chloramphenicol resistance; eryᴿ, erythromycin resistance; NTC, non-target control.

**TABLE 2:** Plasmids used in this study^*b*^

Plasmids	Relevant characteristics	Source or reference
pCM29- P*sarA* P1-GFP	Plasmid carrying *gfp* downstream of the staphylococcal accessory regulator, SarA gene promoter P1, ampᴿ, cmᴿ	(31)
pCM29- P*atl* -GFP	Plasmid carrying *gfp* downstream of *S. aureus* autolysin gene promoter^*a*^, ampᴿ, cmᴿ	This study
pCM29- P*fnbA* -GFP	Plasmid carrying *gfp* downstream of *S. aureus* fibronectin-binding protein A gene promoter^*a*^, ampᴿ, cmᴿ	This study
pCM29-P*coa* -GFP	Plasmid carrying *gfp* downstream of *S. aureus* coagulase gene promoter^*a*^, ampᴿ, cmᴿ	This study
pLOW- P*lac -dcas*9	Plasmid for IPTG-inducible expression of mutated Cas9 (*dcas9*) downstream of *lac* promoter, ampᴿ, eryᴿ.	(11)
pLOW- P*atl* -d*cas*9	Plasmid carrying *dcas9* downstream of *S. aureus* autolysin gene promoter^*a*^, ampᴿ, eryᴿ.	This study
pLOW-P*fnbA* -d*cas*9	Plasmid carrying *dcas9* downstream of *S. aureus* fibronectin-binding protein A gene promoter^*a*^, ampᴿ, eryᴿ.	This study
pLOW-P*coa* -d*cas*9	Plasmid carrying *dcas9* downstream of *S. aureus* coagulase gene promoter^*a*^, ampᴿ, eryᴿ.	This study
pCG248-sgRNA (*pbp1*)	Plasmid carrying sgRNA(*pbp1*) expression cassette, ampᴿ, cmᴿ.	(11)
pCG248-sgRNA (NTC)	Plasmid carrying sgRNA(*luc*) expression cassette, ampᴿ, cmᴿ.	(11)
pVL2336-sgRNA (*coa*)	Plasmid carrying sgRNA(*coa*) expression cassette, ampᴿ, cmᴿ.	This study
pCM29-P*sarA* P1-GFP-sgRNA (*pbp1*)	Plasmid carrying *gfp* downstream of the staphylococcal accessory regulator, SarA gene promoter P1 and sgRNA(*pbp1*) expression cassette, ampᴿ, cmᴿ.	This study
pCM29-P*atl*-GFP-sgRNA (*pbp1*)	Plasmid carrying *gfp* downstream of *S. aureus* autolysin gene promoter^*a*^ and sgRNA(*pbp1*) expression cassette, ampᴿ, cmᴿ.	This study
pCM29-*PfnbA*-GFP -sgRNA(*pbp1*)	Plasmid carrying *gfp* downstream of *S. aureus* fibronectin-binding protein A gene promoter^*a*^ and sgRNA(*pbp1*) expression cassette, ampᴿ, cmᴿ.	This study
pCM29-P*coa*-GFP-sgRNA (*pbp1*)	Plasmid carrying *gfp* downstream of *S. aureus* coagulase gene promoter^*a*^ and sgRNA(*pbp1*) expression cassette, ampᴿ, cmᴿ.	This study
pCM29-P*atl*-GFP-sgRNA (NTC)	Plasmid carrying *gfp* downstream of *S. aureus* autolysin gene promoter^*a*^ and sgRNA(*luc*) expression cassette, ampᴿ, cmᴿ.	This study
pCM29-P*fnbA*-GFP-sgRNA (NTC)	Plasmid carrying *gfp* downstream of *S. aureus* fibronectin-binding protein A gene promoter^*a*^ and sgRNA(*luc*) expression cassette, ampᴿ, cmᴿ.	This study
pCM29-P*coa-*GFP-sgRNA (NTC)	Plasmid carrying *gfp* downstream of *S. aureus* coagulase gene promoter^*a*^ and sgRNA(*luc*) expression cassette, ampᴿ, cmᴿ.	This study
pCM29-P*sarA* P1-GFP-sgRNA (*coa*)	Plasmid carrying *gfp* downstream of the staphylococcal accessory regulator, SarA gene promoter P1, and sgRNA(*coa*) expression cassette, ampᴿ, cmᴿ.	This study

^*a*^ Promoter sequences were selected as the non-coding gap sequence between the gene and its upstream gene.

^*b*^ ampᴿ, ampicillin resistance; cmᴿ, chloramphenicol resistance; eryᴿ, erythromycin resistance; NTC, non-target control.

*E. coli* cells were grown in LB with shaking or on an LB agar plate at 37°C. Ampicillin was used at a final concentration of 100 µg/mL in LB or agar plates for the selection of recombinant plasmid-transformed *E. coli* colonies. *S. aureus* was routinely grown at 37°C on TSA plates or in TSB with shaking (220  rpm). Chloramphenicol (10 µg/mL) was used for the maintenance of the fluorescent reporter plasmids (pCM29- P*sarA* P1-GFP, pCM29-P*atl*-GFP, pCM29-P*fnbA*-GFP, and pCM29-P*coa*-GFP, see next section) in *S. aureus*. Erythromycin (5 µg/mL) and chloramphenicol (10 µg/mL) were used for the selection and maintenance of pLOW and pCM29 backbone plasmids, respectively, in *S. aureus* CRISPRi strains. IPTG was used as 250 µM (final concentration) for *lac*-promoter-induced dCas9 expression in the CRISPRi system. Plasmid pCM29-P*sarA* P1-GFP was provided by Dr. Alexander Horswill (University of Colorado School of Medicine, Colorado, USA) (31). Chemically competent *E. coli* IM08B cells were prepared according to reference (32) and routinely used for the transformation of constructed plasmid according to standard heat shock protocol (33). Chemically competent *E. coli* cells (TOP 10, NEB 5-alpha, NEBExpress I^q^, and NEB 10-beta) were transformed according to the manufacturer’s instructions. *S. aureus* USA 300 LAC was transformed with dCas9 expression CRISPRi plasmid DNA isolated from *S. aureus* RN4220 (30) and fluorescent reporter plasmid DNA isolated from IM08B (34) by electroporation. The preparation of electrocompetent cells and electroporation were performed as described before (35).

### Restriction enzyme digestion and ligation

Restriction enzyme digestion and ligation reactions were performed according to the manufacturer’s instructions (New England Biolabs). Briefly, a prepared restriction digestion reaction mixture containing sample DNA (1 µg), 10× rCutSmart buffer (5 µL), and restriction enzyme (10 units) in a total of 50 µL reaction volume with nuclease-free water was incubated at the optimum temperature for the given restriction enzyme for 2 hours. In this work, restriction digestions with BsmBI were carried out at 55°C, while all other restriction digestions were performed at 37°C. For restriction digestions of destination vectors, 1 µL calf intestinal alkaline phosphatase was added to the reaction after ~2 hours of incubation, followed by a subsequent incubation for ~30 min. Digested DNA was verified using agarose gel electrophoresis and purified either from the agarose gel or directly from the digestion reaction using MagExtractor-PCR and Gel Clean up kit (Toyobo, Osaka, Japan).

Digested vectors and inserts were ligated together using the T4 DNA ligase (New England Biolabs). The components of the reaction were mixed with a molar insert:vector ratio of 3:1. The reaction volume was 20 µL, using 2 µL of the supplied 10× reaction buffer and 1 µL T4 DNA ligase. Ligation reactions were carried out overnight at 16°C. Ligation reactions were stored at −20°C or directly transformed into the desired host.

### Plasmid construction

#### Fluorescent reporter plasmids

Genomic DNA (gDNA) from *S. aureus* USA 300 LAC was isolated using the Wizard Genomic DNA Purification Kit. Using isolated gDNA as a template, PCR was performed using Phusion High-Fidelity DNA Polymerase to amplify the promoter region (non-coding gap sequence between the gene and its upstream gene) of autolysin, SAUSA 300_0955 (*atl*), fibronectin-binding proteins A, SAUSA 300_2441 (*fnbA*), and coagulase, SAUSA 300_0224 (*coa*) with their respective primer sets RM 1/2, RM 3/4, and RM 7/8 (Table S2). The PCR-amplified fragments and the vector pCM29-P*sarA* P1-GFP (31) were both digested with KpnI and PstI. Digested fragments and vector backbone pCM29-GFP were purified after agarose gel electrophoresis and ligated by T4 DNA Ligase (New England Biolabs) to produce the pCM29-P*atl*-GFP, pCM29-P*fnbA*-GFP, and pCM29-P*coa*-GFP construct in which a Green Fluorescent Protein (GFP) reporter is placed under control of promoter P*atl*, P*fnbA*, and P*coa*, respectively. The ligation reaction mix was transformed into *E. coli* IM08B, and cells were plated on LB agar plates containing 100 µg/mL ampicillin, and the correct construct was verified by PCR and sequencing.

#### dCas9 expression low-copy number pLOW-dcas9 plasmid

Gene promoters (P*atl*, P*fnbA*, and P*coa*) were amplified by PCR using previously constructed fluorescent reporter plasmids, pCM29-P*atl*-GFP, pCM29-P*fnbA*-GFP, and pCM29-P*coa*-GFP as the template DNA, respectively, with their primer sets RM 11/12, RM 13/14, and RM 15/16 (Table S2). The PCR amplified fragments and the vector pLOW-P*lac*-dcas9 (11) were both digested with AvrII and SalI. Digested fragments and vector backbone pLOW-dcas9 were purified after agarose gel electrophoresis and ligated by T4 DNA Ligase (New England Biolabs) to produce the pLOW-P*atl*-dcas9, pLOW*-*P*fnbA-*dcas9, and pLOW*-*P*coa-*dcas9 constructs where *dcas9* is placed downstream of each selected gene promoter. The ligation reaction mix was transformed into *E. coli* NEB10-beta, and cells were plated on LB plates containing 100 µg/mL ampicillin, and the correct construct was verified by restriction mapping, PCR, and sequencing. All plasmids used in this study are listed in Table 2.

#### The sgRNA expression high-copy number pCM29-PsarA P1-GFP plasmid

A sgRNA construct, consisting of a 20 nt base-pairing region of targeted genes (*pbp1*, *coa*) or a no-target control (NTC) sequence originally designed to target the luciferase gene (*luc*) along with their Cas9-handle region, located downstream of a synthetic, constitutive promoter P3 in the vector pVL2336-sgRNA/pCG248-sgRNA (9, 11) was cut out using EcoRI. The fragments were ligated into the corresponding sites of vector pCM29-P*sarA* P1-GFP (31), pCM29-P*atl*-GFP, pCM29-P*fnbA*-GFP, and pCM29-P*coa*-GFP by T4 DNA Ligase (New England Biolabs) to construct pCM29-P*sar*AP1-GFP-sgRNA(*pbp1*), pCM29-P*sar*AP1-GFP-sgRNA(*coa*), pCM29-P*atl*-GFP-sgRNA(*pbp1*), pCM29-P*atl*-GFP-sgRNA(NTC), pCM29-P*fnbA*-GFP-sgRNA(*pbp1*), pCM29-P*fnbA*-GFP-sgRNA(NTC), pCM29-P*coa*-GFP-sgRNA(*pbp1*), and pCM29-P*coa*-GFP-sgRNA (NTC). The ligation reaction mix was transformed into *E. coli* IM08B, and cells were plated on LB agar plates containing 100 µg/mL ampicillin, and the correct construct was verified by PCR and sequencing. The sequences of the sgRNA base-pairing regions are given in Table S3.

### Bacterial growth and GFP fluorescence analysis

Bacterial growth (OD_600_) and GFP fluorescence were measured in a microplate assay on a Synergy H1 Hybrid Reader (BioTek). Bacterial cells were cultivated at 37°C overnight with shaking (220 rpm). Cell cultures from *E. coli* WT (IM08B or NEB10-beta), *S. aureus* USA 300 WT, and their fluorescent reporter strains, MR 1–11 (Table 1; Table S1), and fluorescently labeled (MR 16, 27–32) and non-labeled (MR 15) CRISPRi strains (Table 1) were diluted to an OD_600_ of 0.1 in their respective fresh medium (TSB or RPMI+ for *S*. *aureus* and LB for *E. coli* strains). Two microliter of these diluted overnight cultures was inoculated into 298 µL of each respective fresh medium in a 96-well flat-bottom polystyrene tissue culture plate (Falcon). The plate was incubated directly in a Synergy H1 Hybrid Reader (BioTek) microtiter plate reader at 37°C with constant double orbital shaking (400 rpm for 5 s) in between measurements. Optical density (OD_600_) and GFP fluorescence (excitation 479 and emission 520) were measured at 1-hour intervals for up to 24 hours. GFP fluorescence was recorded as relative fluorescence units (RFUs, i.e., relative to the internal standard in the instrument). The OD_600_ and GFP fluorescence values from three biological replicates that were each derived from three technical replicates were averaged and corrected from blank wells containing only medium for each strain.

### RNA purification, reverse transcription, and quantitative PCR

The overnight cultures of coagulase-interfering CRISPRi strains MR 23–26 (Table 1) and their respective non-targeting control strains MR 27, 29, and 31 (Table 1) were diluted to an OD_600_ of 0.1 in fresh TSB or RPMI+ and grown at 37°C for 6 hours. Silencing of the *coa* gene in the P*lac*-based strain MR23 was induced by adding IPTG to a final concentration of 250 µM. Cells from each culture were diluted in PBS (D8537, Sigma) and adjusted to McFarland 0.5 (i.e., about 10^8^ cells/mL). Subsequently, cells were treated with 2 × volume of RNAprotect Bacteria Reagent (QIAGEN) for 10 min at room temperature and collected by centrifugating for 10 min at 5,000 × *g*. Lysostaphin, 1 µg/µL, and lysozyme, 10 µg/µL, were added as final concentration to the suspended solution and incubated at 37°C for 30 minutes for lysis of bacterial cells before RNA isolation. RNA extraction was performed using a RNeasy Mini Kit (QIAGEN). The remaining DNA in the isolated RNA was degraded by DNase I (Sigma-Aldrich). DNA-free RNA was subjected to reverse transcription (RT) with the High-Capacity cDNA Reverse Transcription Kit (Applied Biosystems). RNA extraction, DNase treatment, and RT were performed according to the manufacturer’s direction. Synthesized cDNA solution was 10- and 10,000-fold diluted with RNase-free water, and 2 µL of the diluted cDNA solution was subjected to real-time PCR assay performed with PowerTrack SYBR Green Master Mix (Applied Biosystems) containing 0.5 µM each primer set. After cycling, melt curves analysis was performed between 70°C and 90°C. All quantitative PCR (qPCR) data were analyzed using LightCycler 96 system software version 1.1 (Roche Diagnostics). Relative coagulase expression was calculated according to the 2^−∆∆Ct^ method after normalization by 16S rRNA (rrsA). The real-time PCR primers are listed in Table S2. qPCR was performed in two technical replicates from their three biological replicates.

### Coagulation assays

The overnight cultures of coagulase-interfering CRISPRi strains MR 23–26 (Table 1) and their respective non-targeting control strains MR 27, 29, and 31 (Table 1) were diluted to an OD600 of 0.1 in fresh TSB or RPMI+ and grown at 37°C for 18 hours. Silencing of the *coa* gene in the control strain MR23 [USA300 LAC carrying pLOW-P*lac-dcas*9, pCM29-P*sar*A-P1 *gfp*-sgRNA (*coa*); Table 1] was induced by adding IPTG to a final concentration of 250 µM. Cells from each culture were diluted in PBS (D8537, Sigma-Aldrich) and adjusted to McFarland 0.5 (i.e., about 10^8^ cells/mL). Subsequently, 1 mL of each McFarland-adjusted culture was added to 0.5 mL rabbit plasma (Bio-Rad). The samples were incubated in a water bath at 37°C for 4 hours. The level of coagulation was verified by tipping the tubes to a 45° angle. A negative control (NC) sample contained medium only. A test was considered positive if the plasma in the tube formed a coherent clot. All experiments were repeated three times as independent biological replicates to examine the reproducibility.

## RESULTS

### Design of a vgp-CRISPRi system for *S. aureus*

**Fig 1:**
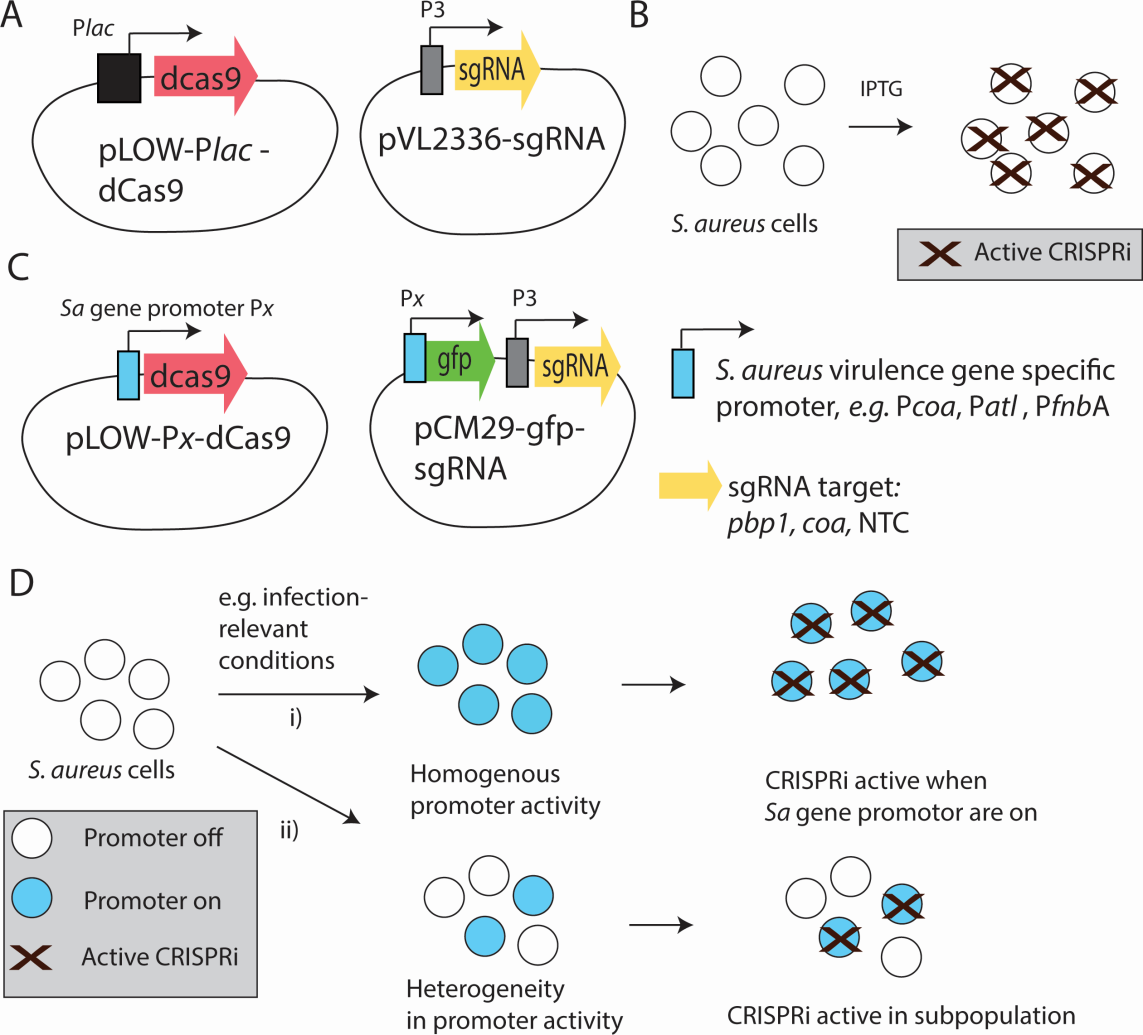
Overview of the IPTG-inducible and the inducer-free, fluorescent reporter-labeled virulence gene promoter-activated CRISPRi system. (**A**) The plasmids used in the IPTG-inducible CRISPRi system. (**B**) Schematic overview of the activation of the CRISPRi system in *S. aureus* cells after IPTG induction. (**C**) Plasmids associated with the vgp-CRISPRi system. dCas9 expression is controlled by a virulence gene promoter (Px) on the pLOW-Px-dCas9 plasmid. The sgRNA is expressed from a pCM29-plasmid under control of the constitutive P3 promoter. This plasmid also carries a *gfp* gene that can be placed under control of a virulence gene promoter of interest. (**D**) Schematic representation of the activation of the vgp-CRISPRi system. The schematic exemplifies the scenario when *dcas9* and *gfp* under control of the same promoter Px. Based on the nature of the promoter and experimental conditions, the promoter may be (i) homogeneously or (ii) heterogeneously activated under the conditions of interest. As a consequence, the CRISPRi system is activated either (i) in the entire cell population or (ii) in a subpopulation only.

In the previously published staphylococcal CRISPRi-system, *dcas9* [*S. pyogenes* (SpydCas9)] expression is controlled by an IPTG-inducible *lac* promoter in the low copy number pLOW-dcas9 plasmid, and sgRNA is expressed by constitutive P3 promoter in the vector pVL2336-sgRNA (or equivalent vector pCG248-sgRNA) (9, 11, 12). To generate an inducer-independent system for *S. aureus*, we replaced the *lac* promoter with *S. aureus*-specific virulence gene promoters in the pLOW plasmid (Fig. 1A through C). We selected well-known virulence gene promoters controlling the expression of genes encoding autolysin (P*atl*), fibronectin-binding protein A (P*fnbA*), and coagulase (P*coa*). Since the activity levels of endogenous virulence gene promoters are dynamic, there is condition-dependent variation as to when the promoter is activated and *dcas9* is expressed, which is a precondition for the activity of the CRISPRi system. To address this question, we incorporated a fluorescent reporter gene into the CRISPRi system by placing *gfp* (into the sgRNA-expressing plasmid) under the control by the same promoter as *dcas9*.

During the generation of these constructs, we encountered technical challenges caused by the unexpected activity of *S. aureus* gene promoters in *E. coli* (Fig. S1) prompting dCas9-related toxicity and plasmid instability in the *E. coli* cloning host IM08B (Fig. S2), a strain that methylates the plasmids to conveniently allow direct transformation into many *S. aureus* lineages including USA300 LAC. These pitfalls were bypassed by identifying *E. coli* NEB10-beta as an alternative cloning host that is less susceptible to dCas9-mediated toxicity (Fig. S2). Isolated plasmids from NEB10-B could then be successfully electroporated into *S. aureus* RN4220 (to enable correct methylation), repurified, and electroporated into the clinical MRSA-isolate USA300 LAC, providing a viable strategy for generating *S. aureus* gene-promoter-driven CRISPRi constructs in *S. aureus*.

### Evaluating dynamic virulence gene promoter activities in *S. aureus*

**Fig 2:**
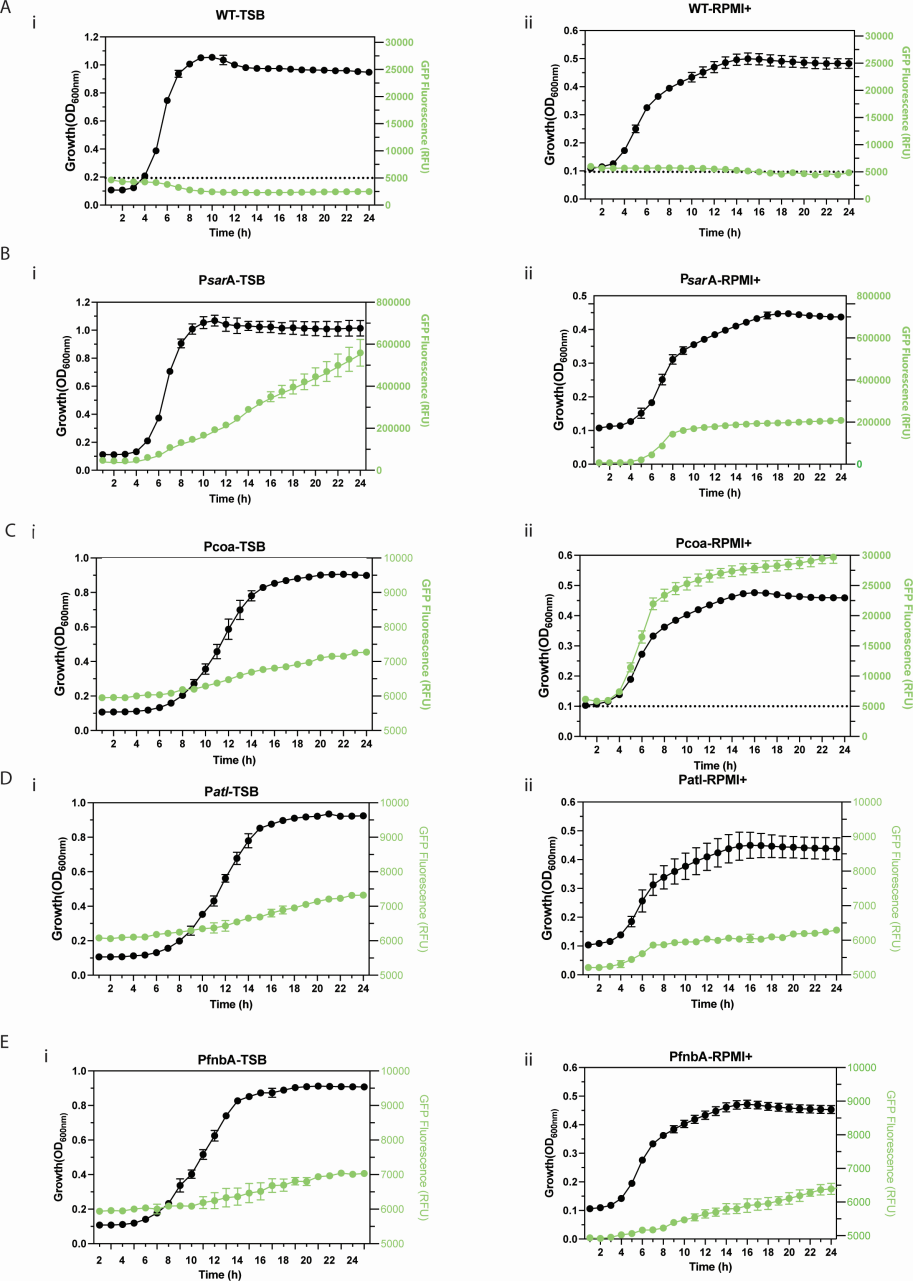
Differential activation of virulence gene promoters in *S. aureus*. Overlays of growth curves (OD_600_) and GFP fluorescence levels (in RFU) of (**A**) *S. aureus* LAC WT and (**B**) p*sar*A-GFP, (**C**) P*coa*-GFP, (**D**) P*atl*-GFP, and (**E**) P*fnb*A-GFP fluorescent reporter strains. Cultures were grown either in (i) TSB or (ii) cell culture medium RPMI+. Data show means ± SD of *n* = 3 biological replicates (each recorded with three technical replicates).

We first evaluated dynamic differences in the selected *S. aureus* promotors under different growth phases and media conditions. We recorded 24-hour-growth curves and fluorescent signals of pCM29-GFP-based fluorescent reporter strains (strains MR 9–11, Table 1) in default bacteriological culture media (TSB) as well as RPMI-1640 supplemented with 10% FBS (RPMI+), a mammalian cell culture medium that mimics the composition of human body fluids (36, 37). Compared to a P*sar*AP1 reporter, all strains showed low levels of fluorescence in TSB that was increasing throughout the exponential and stationary phase (Fig. 2). Interestingly, the P*coa*- reporter strain showed a greater than sixfold increase in GFP signal during exponential growth in RPMI+ indicating the differential activity of these promoters in different environments (Fig. 2). Since P*coa* showed the highest activity levels across the conditions tested, we considered it a suitable promoter for a proof-of-principle experiments with the vgp-CRISPRi system.

### *S. aureus* virulence gene promoter-driven CRISPRi constructs are functional in *S. aureus*

**Fig 3:**
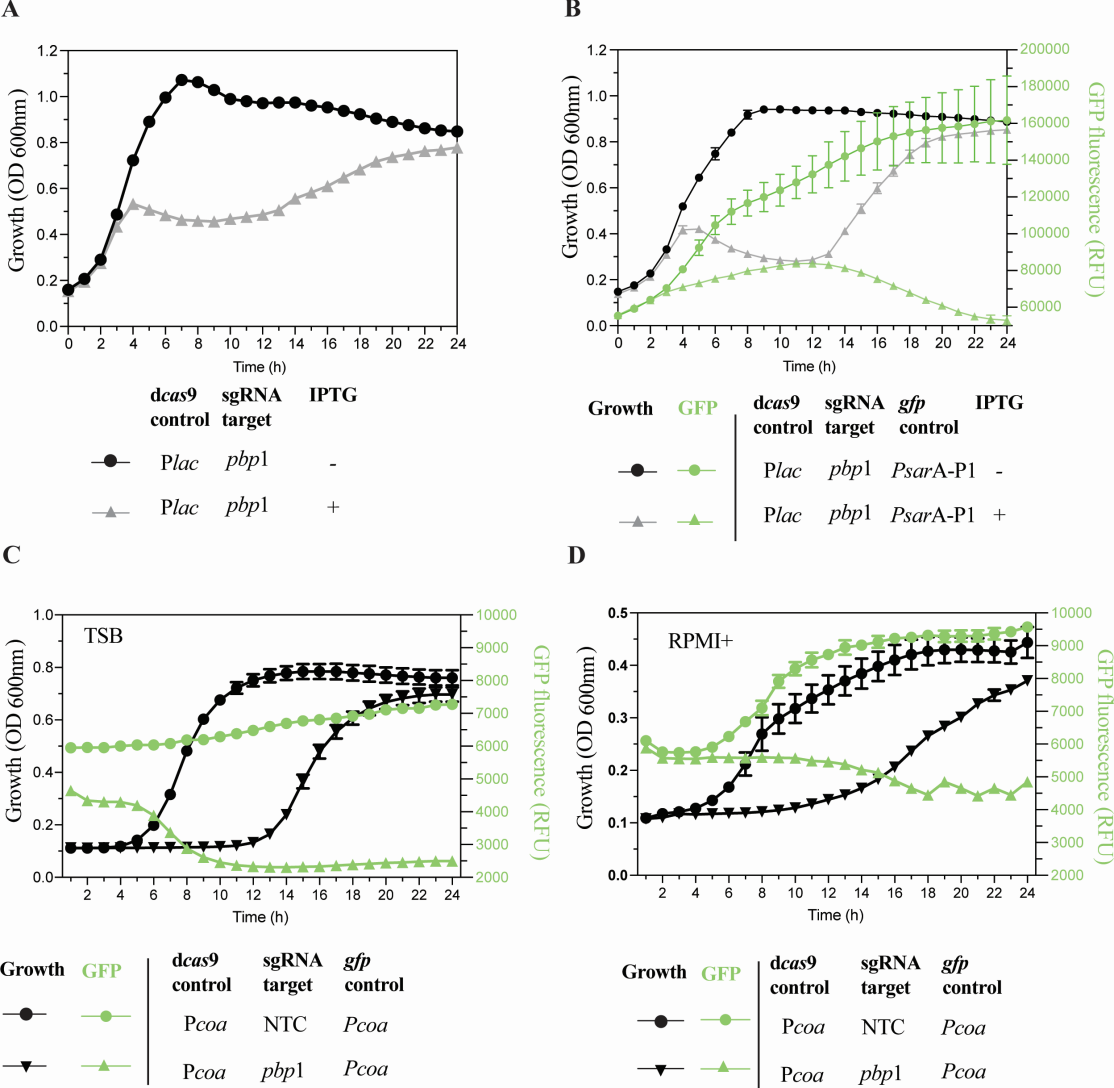
Silencing of *pbp*1 in vgp-CRISPRi induces growth inhibition in *S. aureus* LAC. (**A**) Growth (OD_600_) of the indicated classical pVL2336-based inducible CRISPRi strains grown in TSB in the presence or absence of IPTG (250 µM). (**B**) Overlay of the growth curve (OD_600_) and GFP fluorescence levels (in RFU) of the indicated CRISPRi strains grown in TSB in the presence or absence of IPTG (250 µM) over time. (**C and D**) Overlay of growth (OD_600_) and GFP fluorescence (RFU) over time of the indicated vgp-CRISPRi strains grown in (**C**) TSB or (**D**) RPMI+. *dcas9* and *gfp* expression was controlled by the P*coa* promoter, sgRNAs targeted either the essential peptidoglycan biosynthesis gene *pbp1* (monocistronic) inducing growth inhibition or an NTC sequence derived from the luciferase gene. Data show mean ± SD of *n* = 3 biological replicates (each recorded with three technical replicates).

Classical CRISPRi systems are based on the IPTG-inducible *dcas9* expression pLOW plasmid and sgRNA-target sequence on pVL2236 plasmid (strain MR15; Table 1). We first tested if the pCM29-P*sarA*-P1-*gfp*-based sgRNA plasmids were functional (like the pVL2236 plasmid) in combination with the pLOW plasmid in strain MR16 (Table 1). We chose to target the gene encoding the essential protein PBP1, which has been reported to result in a growth inhibition phenotype upon induction (11). After IPTG induction, a characteristic growth halt for up to 10 hours was observed for both strains, MR15 (Fig. 3A) and MR16 (Fig. 3B). Because strain MR 16 exhibited a similar outcome to MR 15, it indicates that the pCM29-P*sarA*-P1-*gfp*-based sgRNA plasmid is also functional, like the pVL2236 plasmid, in the IPTG-inducible system. Interestingly, growth resumption coincided with a decline in GFP signal (controlled by P*sarA*-P1) after IPTG induction in strain MR16 (Fig. 3B).

Finally, to investigate if *dcas*9 expression under control of virulence gene promoters leads to similar growth arrest when sgRNA is targeting *pbp*1, we generated a CRISPRi strain (MR32; Table 1) carrying pLOW-P*coa*-dcas9 and pCM29-P*coa-gfp*-sgRNA(*pbp1*), in which *dcas9* and *gfp* are controlled by the *coa*-promoter, whereas sgRNA expression is driven by the constitutive P3 promoter (Fig. 1). Indeed, this strain (MR32) also showed pronounced growth inhibition with declined GFP signal in both TSB (Fig. 3C) and RPMI+ (Fig. 3D) when compared to a non-target control strain (MR31; Table 1). CRISPRi constructs where *dcas9* (and *gfp*) was controlled by the P*atl* and P*fnb*A and showed similar temporary growth arrest with declined GFP signal when used in combination with sgRNA (*pbp*1; Fig. S3). Collectively, these observations can be attributed to the emergence of loss-of-function mutations in the CRISPRi system that are commonly observed when targeting essential genes (6, 8, 38).

Of note, the growth patterns of strains with a non-targeted sgRNA of the classical IPTG-inducible system (MR17 and 18; Table 1) and the vgp-CRISPRi system (MR 27, 29, and 31, Table 1) in both TSB and RPMI+ medium were identical, regardless of whether dCas9 expression was induced with IPTG or not, or which promoter was used to control dCas9 expression (Fig. S4). These data suggest that dCas9 expression *per se* does not affect the growth of *S. aureus*. However, compared to the WT, all mentioned CRISPRi strains showed a noticeable growth delay in both TSB and RPMI and a reduction in growth rate in TSB (Fig. S4), which might be attributed to plasmid carriage and growth in the presence of antibiotics selection.

### Virulence gene promoter-driven CRISPRi strains allow specific and robust interference with *coa* gene expression and coagulase function

**Fig 4:**
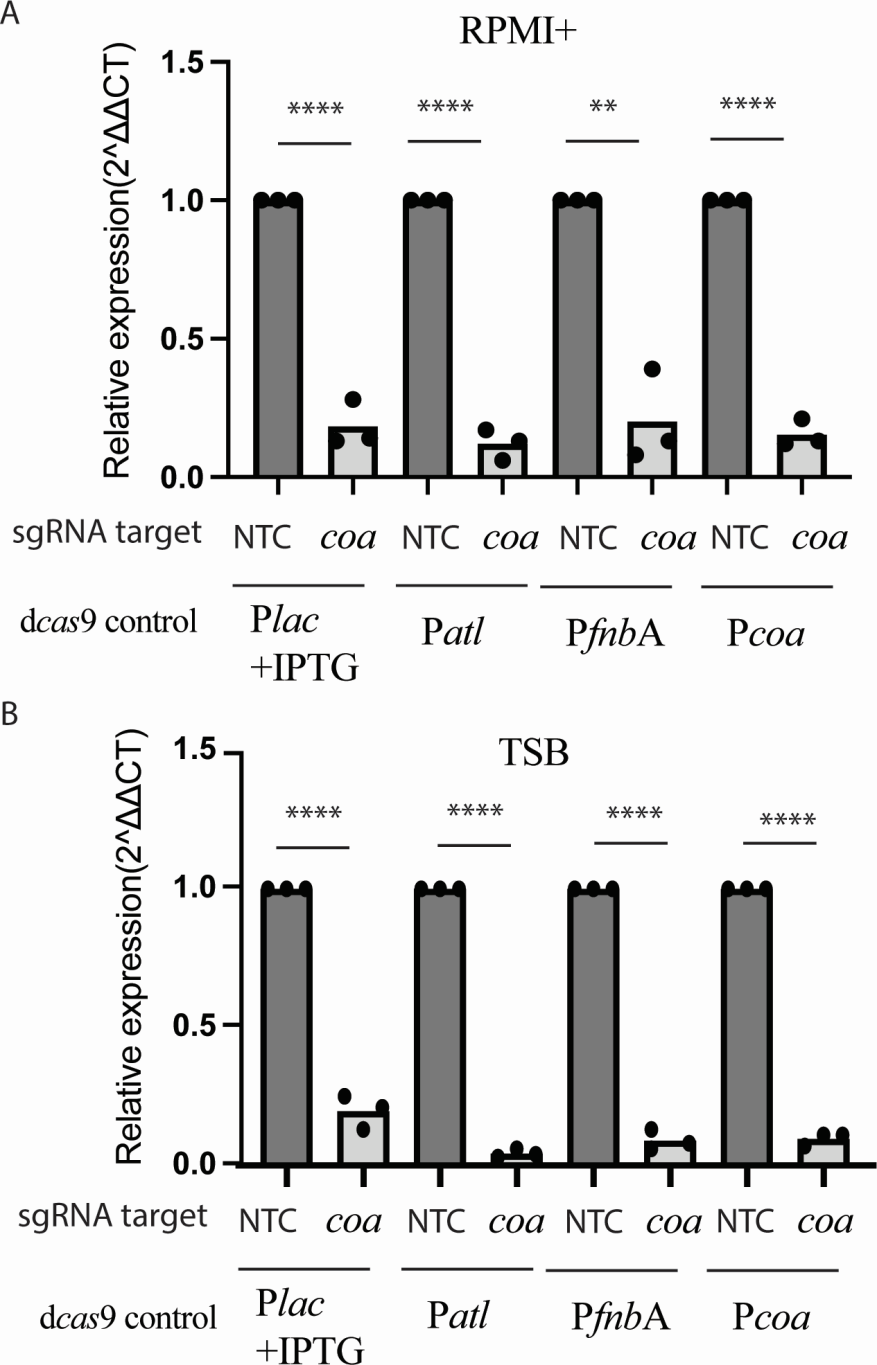
Silencing of *coa* expression by vgp-CRISPRi strains. Repression of *coa* mRNA of the indicated vgp-CRISPRi strains grown in (**A**) TSB or (**B**) RPMI+. The sgRNA target was *coa* or an NTC derived from the luciferase gene, and dCas9 expression was controlled by the P*lac*, P*atl*, P*fnbA*, and P*coa* promoters as indicated. Before total mRNA isolation, strains were grown at 37°C for 6 hours in fresh TSB or RPMI+ with IPTG as indicated. Relative coagulase expression was calculated after normalization by 16S rRNA (rrsA). Data show mean ± SD of *n* = 3 biological replicates (each recorded with two technical replicates). Significance was tested for each construct against its NTC control pair by unpaired, two-tailed Students *t* test.^∗∗^*P* < 0.01 and ^∗∗∗∗^*P* < 0.0001.

**Fig 5:**
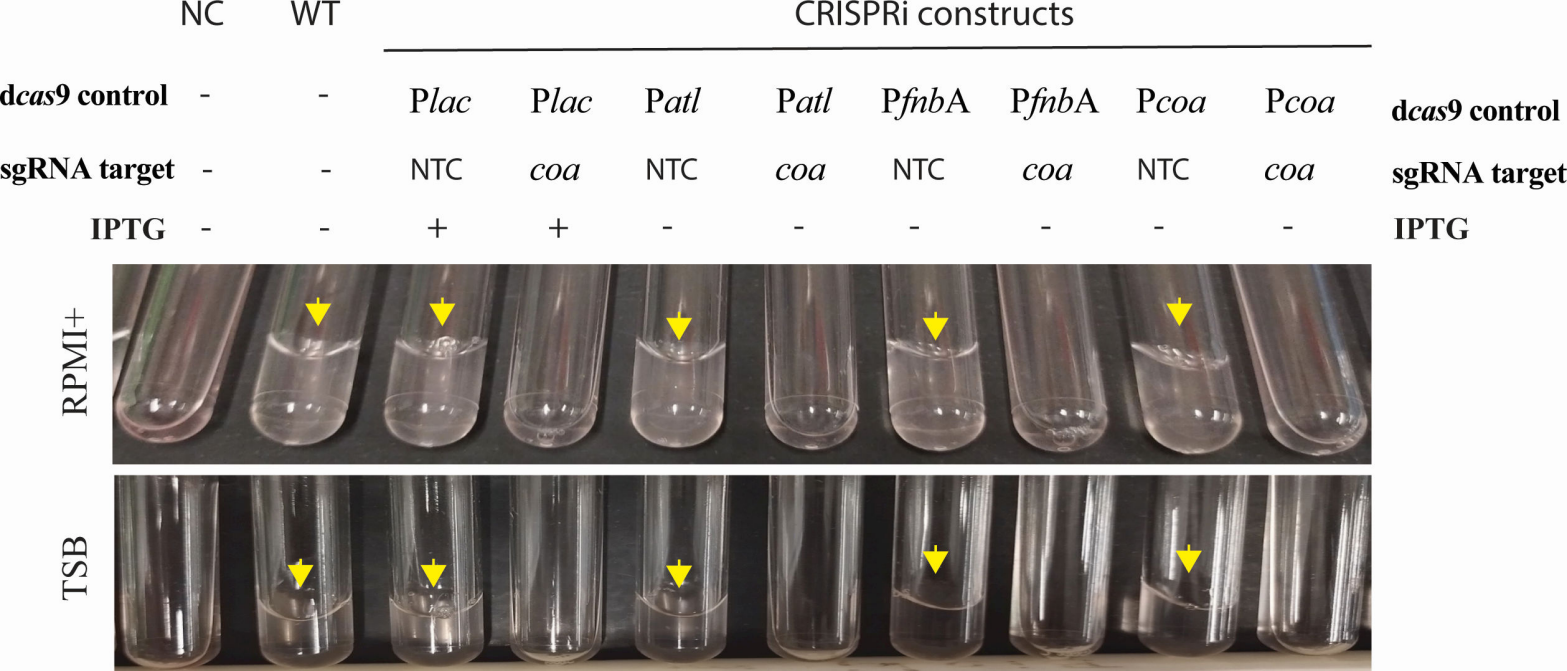
Silencing of *coa* by vgp-CRISPRi strains prevents plasma coagulation. The figure shows photographs of culture tubes 4 hours after adding different CRISPRi strains to rabbit plasma. The tubes were tilted to the side, and coherent clot formation is indicative of coagulation (arrowheads). The sgRNA target was *coa* or an NTC derived from the luciferase gene, dCas9 expression controlled by the P*lac*, P*atl*, P*fnbA*, and P*coa* promoters as indicated. Before the addition of plasma, strains were grown at 37°C for 18 hours in fresh TSB or RPMI+ with or without IPTG as indicated. The figure is representative for three biological replicates. The NC contained medium (RPMI+ or TSB) with rabbit plasma suspension, and WT represents *S.aureus* USA 300 LAC strain.

Finally, we aimed to demonstrate that vgp-CRISPRi is suitable to interfere with specific virulence-associated phenotypes. We focused on coagulase, which converts fibrinogen to fibrin thus inducing clotting of blood plasma. We constructed CRISPRi strains with *coa* as the sgRNA target and dCas9 expression under the control of the P*lac*, P*atl*, P*fnb*A, and P*coa* promoters, respectively (MR23–26; Table 1). We first analyzed the effect on gene expression. qPCR analysis revealed that vgp-CRISPRi strains in which *dcas9* expression was driven by either P*atl*, P*fnb*A, or P*coa P* showed reduced *coa* levels that were similar to those achieved with the IPTG inducible system in both TSB and RPMI+ (Fig. 4). Having confirmed the effect on gene expression, we proceeded to test if silencing of the *coa* gene was sufficient to interfere with the biological function of coagulase. While the addition of WT bacteria to rabbit plasma induced visible clots after 4 hours, no clotting was observed for either of these inducer-free constructs (nor the IPTG-inducible CRISPRi strains with IPTG induction) suggesting successful inhibition of coagulation by knock-down of the *coa* gene (Fig. 5). Non-target controls showed coagulation levels comparable to the WT. The same outcome was achieved regardless of whether bacteria were originally cultivated in TSB or RPMI+, suggesting that all tested promoters were sufficiently activated for the functioning of vgp-CRISPRi system.

## DISCUSSION

Here, we have developed an alternative CRISPRi strategy, in which dCas9 expression is controlled through specific *S. aureus* virulence gene promoters, and we have shown that it is functional in *S. aureus*. The phenotypes induced by gene silencing (temporary growth arrest by silencing of *pbp*1, interference with *coa* expression, and *coa*-dependent coagulation of plasma) using the vgp-CRISPRi system were qualitatively and quantitatively similar to effects achieved with the classical IPTG-inducible system. Since activity levels of the investigated promoters (P*coa*, P*atl*, and P*fnb*A) are low in comparison to constitutive promoter (P*sarA* P1) and variable across growth stages and media conditions, the similar outcome suggests that relatively low levels of dCas9 expression are sufficient for robust activation of the CRISPRi system across the conditions tested. These data position the vgp-CRISPRi system as an alternative for studying gene function in experimental systems for which the use of exogenous inducers such as IPTG or antibiotics has pharmacokinetic limitations or induces off-target effects.

During the construction of this vgp-CRISPRi system, we encountered unexpected dCas9 toxicity and plasmid instability issues during cloning in *E. coli*. We speculate that the instability of the dCas9-expression plasmids in *E. coli* IM08B is caused by the expression of dCas9 from *S. aureus* gene promoters in *E. coli*. This leads to toxic levels of dCas9 for this strain, which results in the selection of loss-of-function mutations in the CRISPRi plasmids. Here, we were able to avoid dCas9 toxicity-dependent issues in plasmid stability by testing alternative cloning host strains and established a viable cloning strategy using NEB-10beta. The need to transform the resulting CRISPRi plasmids first into *S. aureus* RN4220 makes this approach more time-consuming compared to the use of an *E. coli* methylation host such IM08B that allows for direct transformation into relevant *S. aureus* strains. We, therefore, suggest that a close examination of dCas9-toxicity of other strains of the IMxxB series (34) may identify suitable strains that may expedite vgp-CRISPRi strain generation. Consistent with previous studies (9, 11), we did not observe a toxic effect related to dCas9 expression in *S. aureus*. However, compared to the WT all strains carrying the CRISPRi plasmids (regardless of dCas9 expression) showed delayed growth and a reduced growth rate in specific media. This highlights the importance of NTC controls to avoid misinterpretations and false attributions of such growth defects to repression of target genes.

Classical CRISPRi setups are used to study a “gene-of-interest” through silencing with an appropriate sgRNA, whereas the promoter that controls dCas9 is merely used as means-to-an-end. The addition of an external inducer will yield a homogeneous activation of the classical CRISPRi system in bulk populations not considering the complexity associated with phenotypic heterogeneity within a cell population. Since it is emerging that the expression of many virulence genes indeed is heterogeneous across cells within isogenic populations (39–41), the development of vgp-CRISPRi promises to target and restrict the gene knock-down to specific subpopulations in which a “promoter-of-interest” is active. Thus, the vgp-CRISPRi system may hold promise for dissecting the role of isogenic bacterial subpopulations.

To enable visual tracking of relevant promoter activities over time and across cell populations, we incorporated a GFP reporter. The fluorescent reporter gene was included in the pCM29-based sgRNA plasmid where it was placed under control of the same virulence gene promoter that expresses dCas9 on the pLOW plasmid. Thus, both dCas9 and GFP will be expressed when the virulence gene promoter is active. However, when the sgRNA targets the essential *pbp*1, the GFP signal declines once cells overcome the initial CRISPRi-induced growth arrest. We speculate that the selective pressure of interfering with essential gene-induced mutations in the plasmid that leads to both the inactivation of the CRISPRi system and—as a bystander—the GFP reporter signal.

This system may be developed further by also replacing the constitutive P3 promoter that controls the sgRNA with an additional endogenous gene promoter, effectively creating an AND-gate scenario where activation of the CRISPRi system would require the simultaneous activity of two endogenous promoters. We believe that the adaptability and flexibility of the vgp-CRISPRi system will enable diverse application studies aimed at deciphering the functional dynamics of bacterial cell populations at the host-pathogen interface and provide a pathway for the functional engineering of bacterial cell populations.

## SUPPLEMENTAL MATERIAL

Supplemental figures and tables spectrum.00602-24-s0001.pdf Fig. S1-S4; Tables S1-S3.

ASM does not own the copyrights to Supplemental Material that may be linked to, or accessed through, an article. The authors have granted ASM a non-exclusive, world-wide license to publish the Supplemental Material files. Please contact the corresponding author directly for reuse.

## References

[1] Qi LS, Larson MH, Gilbert LA, Doudna JA, Weissman JS, Arkin AP, Lim WA. 2013. Repurposing CRISPR as an RNA-guided platform for sequence-specific control of gene expression. Cell 152:1173–1183. doi:10.1016/j.cell.2013.02.022 23452860 PMC3664290

[2] Kim SK, Seong W, Han GH, Lee DH, Lee SG. 2017. CRISPR interference-guided multiplex repression of endogenous competing pathway genes for redirecting metabolic flux in Escherichia coli. Microb Cell Fact 16:188. doi:10.1186/s12934-017-0802-x 29100516 PMC5670510

[3] Santos-Moreno J, Tasiudi E, Stelling J, Schaerli Y. 2020. Multistable and dynamic CRISPRi-based synthetic circuits. Nat Commun 11:2746. doi:10.1038/s41467-020-16574-1 32488086 PMC7265303

[4] Rueff A-S, van Raaphorst R, Aggarwal SD, Santos-Moreno J, Laloux G, Schaerli Y, Weiser JN, Veening J-W. 2023. Synthetic genetic oscillators demonstrate the functional importance of phenotypic variation in pneumococcal-host interactions. Nat Commun 14:7454. doi:10.1038/s41467-023-43241-y 37978173 PMC10656556

[5] Peters JM, Colavin A, Shi H, Czarny TL, Larson MH, Wong S, Hawkins JS, Lu CHS, Koo B-M, Marta E, Shiver AL, Whitehead EH, Weissman JS, Brown ED, Qi LS, Huang KC, Gross CA. 2016. A comprehensive, CRISPR-based functional analysis of essential genes in bacteria. Cell 165:1493–1506. doi:10.1016/j.cell.2016.05.003 27238023 PMC4894308

[6] Liu X, Gallay C, Kjos M, Domenech A, Slager J, van Kessel SP, Knoops K, Sorg RA, Zhang J-R, Veening J-W. 2017. High-throughput CRISPRi phenotyping identifies new essential genes in Streptococcus pneumoniae. Mol Syst Biol 13:931. doi:10.15252/msb.20167449 28490437 PMC5448163

[7] Liu X, Kimmey JM, Matarazzo L, de Bakker V, Van Maele L, Sirard J-C, Nizet V, Veening J-W. 2021. Exploration of bacterial bottlenecks and Streptococcus pneumoniae pathogenesis by CRISPRi-Seq. Cell Host Microbe 29:107–120. doi:10.1016/j.chom.2020.10.001 33120116 PMC7855995

[8] de Bakker V, Liu X, Bravo AM, Veening J-W. 2022. CRISPRi-seq for genome-wide fitness quantification in bacteria. Nat Protoc 17:252–281. doi:10.1038/s41596-021-00639-6 34997243

[9] Liu X, de Bakker V, Heggenhougen MV, Mårli MT, Frøynes AH, Salehian Z, Porcellato D, Morales Angeles D, Veening J-W, Kjos M. 2024. Genome-wide CRISPRi screens for high-throughput fitness quantification and identification of determinants for dalbavancin susceptibility in Staphylococcus aureus. mSystems 9:e0128923. doi:10.1128/msystems.01289-23 38837392 PMC11265419

[10] Reed P, Sorg M, Alwardt D, Serra L, Veiga H, Schäper S, Pinho MG. 2024. A CRISPRi-based genetic resource to study essential Staphylococcus aureus genes. mBio 15:e0277323. doi:10.1128/mbio.02773-23 38054745 PMC10870820

[11] Stamsås GA, Myrbråten IS, Straume D, Salehian Z, Veening JW, Håvarstein LS, Kjos M. 2018. CozEa and CozEb play overlapping and essential roles in controlling cell division in Staphylococcus aureus. Mol Microbiol 109:615–632. doi:10.1111/mmi.13999 29884993

[12] Myrbråten IS, Wiull K, Salehian Z, Håvarstein LS, Straume D, Mathiesen G, Kjos M. 2019. CRISPR interference for rapid knockdown of essential cell cycle genes in Lactobacillus plantarum. mSphere 4:e00007-19. doi:10.1128/mSphere.00007-19 30894429 PMC6429040

[13] Costello A, Lao NT, Gallagher C, Capella Roca B, Julius LAN, Suda S, Ducrée J, King D, Wagner R, Barron N, Clynes M. 2019. Leaky expression of the TET-on system hinders control of endogenous miRNA abundance. Biotechnol J 14:e1800219. doi:10.1002/biot.201800219 29989353

[14] Bateman BT, Donegan NP, Jarry TM, Palma M, Cheung AL. 2001. Evaluation of a tetracycline-inducible promoter in Staphylococcus aureus in vitro and in vivo and its application in demonstrating the role of sigB in microcolony formation. Infect Immun 69:7851–7857. doi:10.1128/IAI.69.12.7851-7857.2001 11705967 PMC98881

[15] Moullan N, Mouchiroud L, Wang X, Ryu D, Williams EG, Mottis A, Jovaisaite V, Frochaux MV, Quiros PM, Deplancke B, Houtkooper RH, Auwerx J. 2015. Tetracyclines disturb mitochondrial function across eukaryotic models: a call for caution in biomedical research. Cell Rep 10:1681–1691. doi:10.1016/j.celrep.2015.02.034 25772356 PMC4565776

[16] Ahler E, Sullivan WJ, Cass A, Braas D, York AG, Bensinger SJ, Graeber TG, Christofk HR. 2013. Doxycycline alters metabolism and proliferation of human cell lines. PLoS One 8:e64561. doi:10.1371/journal.pone.0064561 23741339 PMC3669316

[17] Chen W, Zhang Y, Yeo WS, Bae T, Ji Q. 2017. Rapid and efficient genome editing in Staphylococcus aureus by using an engineered CRISPR/Cas9 system. J Am Chem Soc 139:3790–3795. doi:10.1021/jacs.6b13317 28218837

[18] Dong X, Jin Y, Ming D, Li B, Dong H, Wang L, Wang T, Wang D. 2017. CRISPR/dCas9-mediated inhibition of gene expression in Staphylococcus aureus. J Microbiol Methods 139:79–86. doi:10.1016/j.mimet.2017.05.008 28522389

[19] Lee YJ, Hoynes-O’Connor A, Leong MC, Moon TS. 2016. Programmable control of bacterial gene expression with the combined CRISPR and antisense RNA system. Nucleic Acids Res 44:2462–2473. doi:10.1093/nar/gkw056 26837577 PMC4797300

[20] Rock JM, Hopkins FF, Chavez A, Diallo M, Chase MR, Gerrick ER, Pritchard JR, Church GM, Rubin EJ, Sassetti CM, Schnappinger D, Fortune SM. 2017. Programmable transcriptional repression in mycobacteria using an orthogonal CRISPR interference platform. Nat Microbiol 2:16274. doi:10.1038/nmicrobiol.2016.274 28165460 PMC5302332

[21] Cui L, Vigouroux A, Rousset F, Varet H, Khanna V, Bikard D. 2018. A CRISPRi screen in E. coli reveals sequence-specific toxicity of dCas9. Nat Commun 9:1912. doi:10.1038/s41467-018-04209-5 29765036 PMC5954155

[22] Cho S, Choe D, Lee E, Kim SC, Palsson B, Cho BK. 2018. High-level dCas9 expression induces abnormal cell morphology in Escherichia coli. ACS Synth Biol 7:1085–1094. doi:10.1021/acssynbio.7b00462 29544049

[23] Misra CS, Bindal G, Sodani M, Wadhawan S, Kulkarni S, Gautam S, Mukhopadhyaya R, Rath D. 2019. Determination of Cas9/dcas9 associated toxicity in microbes. bioRxiv. doi:10.1101/848135

[24] Rostain W, Grebert T, Vyhovskyi D, Pizarro PT, Tshinsele-Van Bellingen G, Cui L, Bikard D. 2023. Cas9 off-target binding to the promoter of bacterial genes leads to silencing and toxicity. Nucleic Acids Res 51:3485–3496. doi:10.1093/nar/gkad170 36929199 PMC10123097

[25] Moormeier DE, Bose JL, Horswill AR, Bayles KW. 2014. Temporal and stochastic control of Staphylococcus aureus biofilm development. mBio 5:e01341-14. doi:10.1128/mBio.01341-14 25316695 PMC4205790

[26] García-Betancur JC, Goñi-Moreno A, Horger T, Schott M, Sharan M, Eikmeier J, Wohlmuth B, Zernecke A, Ohlsen K, Kuttler C, Lopez D. 2017. Cell differentiation defines acute and chronic infection cell types in Staphylococcus aureus. Elife 6:e28023. doi:10.7554/eLife.28023 28893374 PMC5595439

[27] DelMain EA, Moormeier DE, Endres JL, Hodges RE, Sadykov MR, Horswill AR, Bayles KW. 2020. Stochastic expression of Sae-dependent virulence genes during Staphylococcus aureus biofilm development is dependent on SaeS. mBio 11:e03081-19. doi:10.1128/mBio.03081-19 31937649 PMC6960292

[28] Staats A, Burback PW, Casillas-Ituarte NN, Li D, Hostetler MR, Sullivan A, Horswill AR, Lower SK, Stoodley P. 2023. In vitro staphylococcal aggregate morphology and protection from antibiotics are dependent on distinct mechanisms arising from postsurgical joint components and fluid motion. J Bacteriol 205:e0045122. doi:10.1128/jb.00451-22 36951588 PMC10127631

[29] Voyich JM, Braughton KR, Sturdevant DE, Whitney AR, Saïd-Salim B, Porcella SF, Long RD, Dorward DW, Gardner DJ, Kreiswirth BN, Musser JM, DeLeo FR. 2005. Insights into mechanisms used by Staphylococcus aureus to avoid destruction by human neutrophils. J Immunol 175:3907–3919. doi:10.4049/jimmunol.175.6.3907 16148137

[30] Kreiswirth BN, Löfdahl S, Betley MJ, O’Reilly M, Schlievert PM, Bergdoll MS, Novick RP. 1983. The toxic shock syndrome exotoxin structural gene is not detectably transmitted by a prophage. Nature 305:709–712. doi:10.1038/305709a0 6226876

[31] Pang YY, Schwartz J, Thoendel M, Ackermann LW, Horswill AR, Nauseef WM. 2010. agr-dependent interactions of Staphylococcus aureus USA300 with human polymorphonuclear neutrophils. J Innate Immun 2:546–559. doi:10.1159/000319855 20829608 PMC2982852

[32] Chang Y, Chau WY, Landas A, Pang Y. 2017. Preparation of calcium competent Escherichia coli and heat-shock transformation. JEMI methods 1:22–25.

[33] Froger A, Hall JE. 2007. Transformation of plasmid DNA into E. coli using the heat shock method. J Vis Exp 6:253. doi:10.3791/253 PMC2557105 18997900

[34] Monk IR, Tree JJ, Howden BP, Stinear TP, Foster TJ. 2015. Complete bypass of restriction systems for major Staphylococcus aureus lineages. mBio 6:e00308-15. doi:10.1128/mBio.00308-15 26015493 PMC4447248

[35] Löfblom J, Kronqvist N, Uhlén M, Ståhl S, Wernérus H. 2007. Optimization of electroporation-mediated transformation: Staphylococcus carnosus as model organism. J Appl Microbiol 102:736–747. doi:10.1111/j.1365-2672.2006.03127.x 17309623

[36] Chandra J, Patel JD, Li J, Zhou G, Mukherjee PK, McCormick TS, Anderson JM, Ghannoum MA. 2005. Modification of surface properties of biomaterials influences the ability of Candida albicans to form biofilms. Appl Environ Microbiol 71:8795–8801. doi:10.1128/AEM.71.12.8795-8801.2005 16332875 PMC1317330

[37] Wijesinghe G, Dilhari A, Gayani B, Kottegoda N, Samaranayake L, Weerasekera M. 2019. Influence of laboratory culture media on in vitro growth, adhesion, and biofilm formation of Pseudomonas aeruginosa and Staphylococcus aureus. Med Princ Pract 28:28–35. doi:10.1159/000494757 30352435 PMC6558334

[38] Zhao H, Sun Y, Peters JM, Gross CA, Garner EC, Helmann JD. 2016. Depletion of undecaprenyl pyrophosphate phosphatases disrupts cell envelope biogenesis in Bacillus subtilis. J Bacteriol 198:2925–2935. doi:10.1128/JB.00507-16 27528508 PMC5055597

[39] Nuss AM, Schuster F, Roselius L, Klein J, Bücker R, Herbst K, Heroven AK, Pisano F, Wittmann C, Münch R, Müller J, Jahn D, Dersch P. 2016. A precise temperature-responsive bistable switch controlling Yersinia virulence. PLoS Pathog 12:e1006091. doi:10.1371/journal.ppat.1006091 28006011 PMC5179001

[40] Arnfinnsdottir NB, Bjørkøy AV, Lale R, Sletmoen M. 2016. Heterogeneity in GFP expression in isogenic populations of P. putida KT2440 investigated using flow cytometry and bacterial microarrays. RSC Adv 6:36198–36206. doi:10.1039/C5RA23757B

[41] Surve MV, Bhutda S, Datey A, Anil A, Rawat S, Pushpakaran A, Singh D, Kim KS, Chakravortty D, Banerjee A. 2018. Heterogeneity in pneumolysin expression governs the fate of Streptococcus pneumoniae during blood-brain barrier trafficking. PLoS Pathog 14:e1007168. doi:10.1371/journal.ppat.1007168 30011336 PMC6062133

