# Supplementary material for: Development of an inducer-free, virulence gene promoter-controlled, and fluorescent reporter-labeled CRISPR interference system in Staphylococcus aureus
Source: Microbiol Spectr. 2024 Aug 20;12(10):e00602-24. doi: 10.1128/spectrum.00602-24 (PMC11448056; doi:10.1128/spectrum.00602-24)
Supplement: Supplemental figures and tables — Fig. S1-S4; Tables S1-S3. [file spectrum.00602-24-s0001.pdf]

Supplementary Information

Development of an inducer-free, virulence gene promoter controlled, and fluorescent reporter labeled CRISPR interference system in *Staphylococcus aureus*

Roni Miah[1,*], Mona Johannessen[1], Morten Kjos[2], and Christian S. Lentz[1,*]

[1]Department of Medical Biology and Centre for New Antibacterial Strategies (CANS), UiT-The Arctic University of Norway, 9019, Tromsø, Norway

[2]Faculty of Chemistry, Biotechnology and Food Science, Norwegian University of Life Sciences, Ås, Norway

*To whom correspondence should be addressed: Roni Miah (roni.miah@uit.no) and Christian Lentz (Christian.s.lentz@uit.no)

TABLE OF CONTENTS

Supplementary Figure S1

Supplementary Figure S2

Supplementary Figure S3

Supplementary Figure S4

Supplementary Table S1

Supplementary Table S2

Supplementary Table S3

Supplementary Information

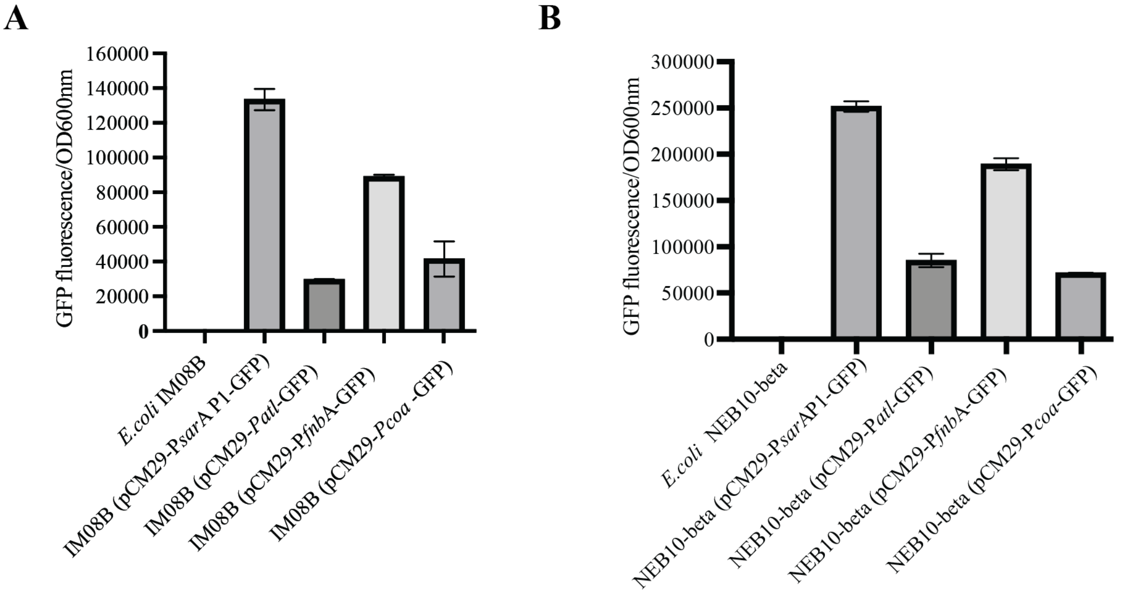

FIG S1 *S. aureus* gene promoters are functional in *E. coli*.

S. aureus gene promoter-based fluorescent reporter plasmids (pCM29-P*sarA* P1-GFP, pCM29-P*atl*-GFP, pCM29-P*fnbA*-GFP, and pCM29-P*coa*-GFP) were transformed into **(A)** *E.coli* IM08B and **(B)** NEB 10-beta strains to check the level of functionality of each gene promoter by GFP fluorescence intensity measurement. GFP fluorescence data was normalized to the OD600 value of the corresponding sample. The *E.coli* strains without fluorescent reporter plasmid are included as controls. Bars show means ± standard deviation of n=3 biological replicates (each recorded with 3 technical replicates).

Supplementary Information

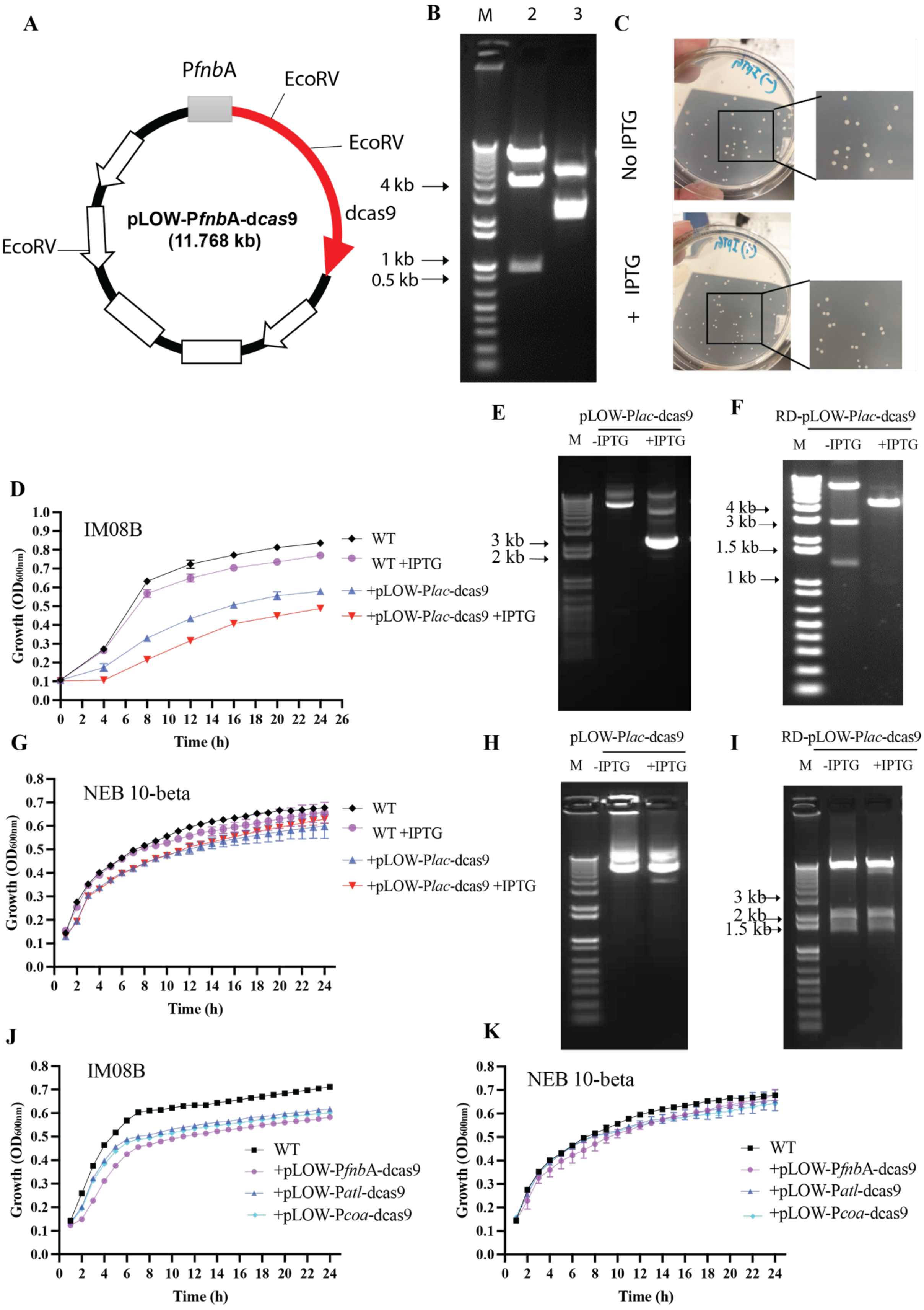

FIG S2 dCas9 toxicity confers plasmid instability in *E.coli* IM08B, but not in NEB 10-beta (A) Schematic diagram of a representative *dcas9* expression plasmid (pLOW-P*fnbA*-

*dcas*9). **(B)** Restriction mapping (*Eco*RV digestion) was performed on isolated pLOW-P*fnbA*-d*cas*9 plasmids from single colonies to check for the correct construct having three fragments: 930 bp, 3.737 kb, and 6.93 kb. Restriction digest of plasmid from *E. coli* IM08B resulted in fragments of unexpected sizes (B, lane 3) while restriction digest of the plasmid from *E.coli* NEB 10-beta had fragments of expected sizes (B, lane 2). **(C)** Photographs of *E. coli* IM08B transformants with pLOW-P*lac*-dcas9 that were grown overnight under non-dCas9 inducing conditions (top image; LB agar with ampicillin) and dCas9-inducing condition (bottom image; LB agar containing ampicillin and 250 µM IPTG) where small-colony phenotypes indicate cell toxicity. (**D**) Growth curves of IM08B (WT) and IM08B (pLOW-P*lac*-dcas9) transformants in the presence or absence of IPTG (250 µM). (**E, F**) Agarose-gel electrophoresis of (**E**) full length pLOW-P*lac*-dcas9 plasmids isolated from IM08B in non-dCas9 expressed condition (no IPTG induction) and highly expressed condition (250µM IPTG induction) and (**F**) the same plasmids after restriction digest (RD) revealing modifications in the plasmid under dCas9-inducing conditions. Double-digestion of the pLOW-P*lac*-dcas9 with PstI and EcoRI was expected to give three different fragments: 1.37 kb, 2.78 kb, and 7.17 kb, which was the case in the absence but not in the presence of IPTG, suggesting dCas9 induced toxicity confer plasmid instability. M= marker: 1.0 kb Plus DNA Ladder). **G**) Growth curves of NEB 10-beta WT and NEB 10-beta (pLOW-P*lac*-dcas9) transformants in the presence or absence of IPTG (250 µM) show little to no signs of dCas9 related toxicity. (**H, I**) Agarose-gel electrophoresis of (**H**) full length pLOW-P*lac*-dcas9 plasmids isolated from NEB-10 beta in non-dCas9 expressed condition (no IPTG induction) and highly expressed condition (250 µM IPTG induction) and (**I**) of the same plasmids after *Kpn*I restriction digest shows that the plasmids isolated in dCas9-inducing and non-inducing conditions are identical. Single-digestion of the pLOW-P*lac*-dcas9 with *Kpn*I was expected to give three different fragments: 1.39 kb, 1.79 kb, and 8.13 kb. M= marker 1.0 kb Plus DNA Ladder. **(J, K)** Growth analysis of the *E. coli* strains containing *S. aureus* gene promoter-derived dCas9 expression plasmids (pLOW- P*atl* -dacs9, pLOW- P*fnbA*-dacs9 and pLOW- P*coa* -dacs9), (**J**) *E. coli* IM08B and (**K**) NEB 10-beta give evidence of dCas9-related toxicity in IM08B, but not in NEB 10-beta.

Supplementary Information

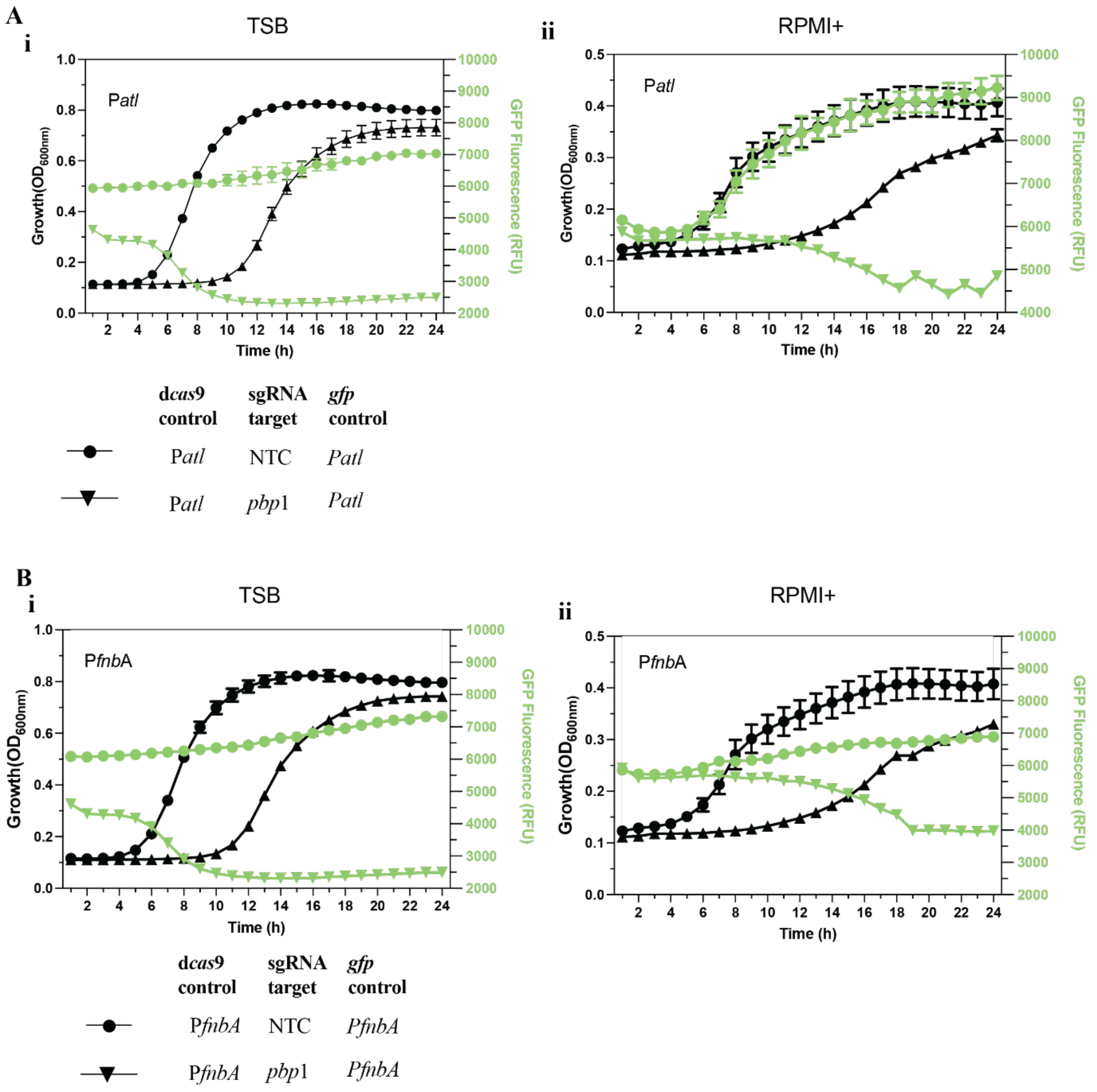

FIG S3 Activation of the vgp-CRISPRi by P*atl* and P*fnb*A in *S. aureus* USA 300 LAC (A, B). i) Growth curves (OD_{600}) and GFP fluorescence over the growth of the indicated vgp-CRISPRi strains (MR 27-30, Table 2) in either TSB (**A**i, **B**i) or RPMI+ (**A**ii, **B**ii). In the strains tested *dcas9* and *gfp* are under the control of P*atl* (**A**) and P*fnbA* (**B**). The sgRNA target genes are indicated in the figure. Data shows means ± standard deviation of n=3 biological replicates (each recorded with 3 technical replicates).

Supplementary Information

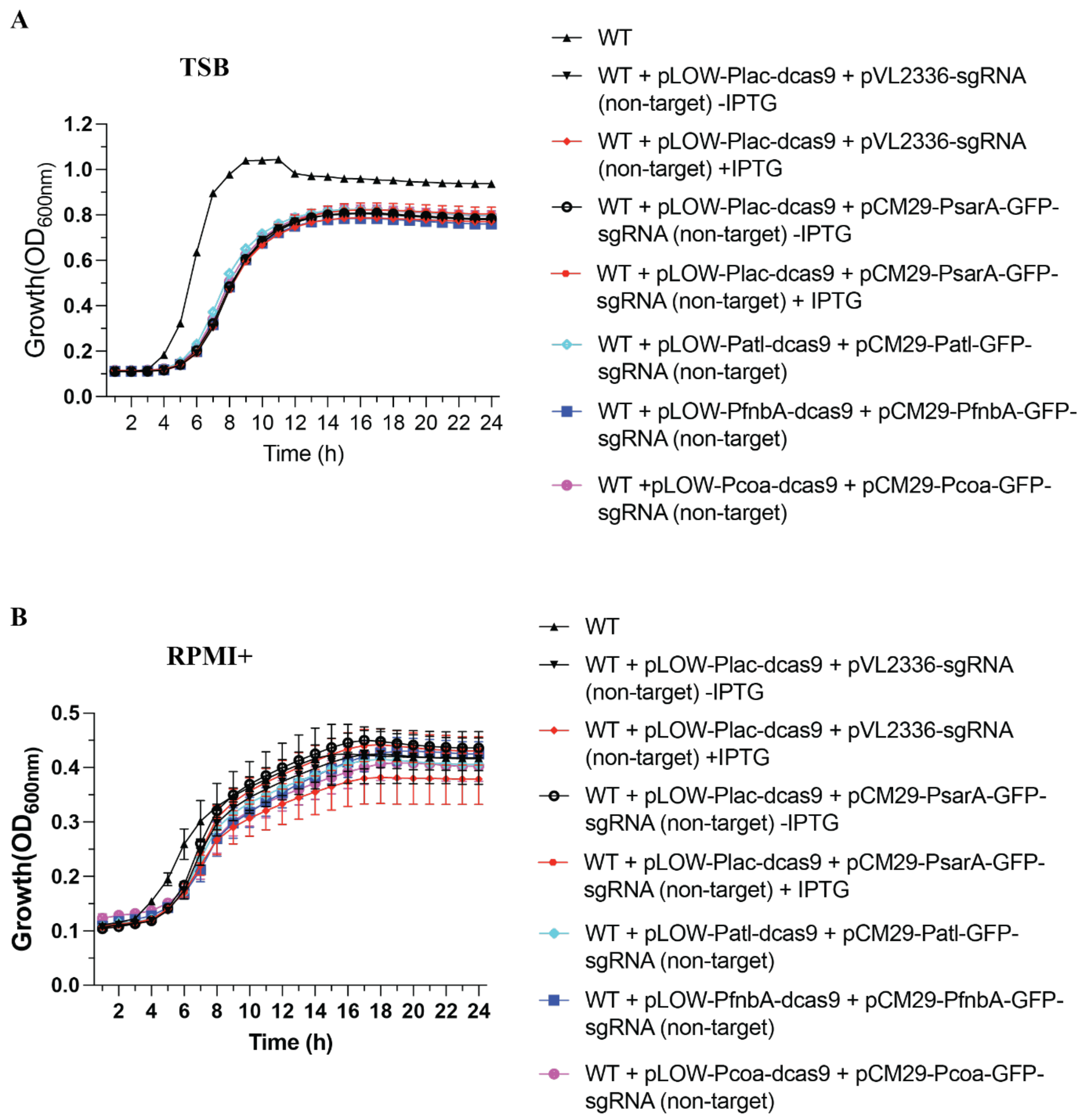

FIG S4 dCas9 expression effect on *S. aureus* growth.

(A,B) Growth curves of WT (*S. aureus* USA 300 LAC*)* and its non-targeting classical CRISPRi strains (MR 17, 18, Table 2), and vgp-CRISPRi strains (MR 27, 29, 31, Table 2) in (**A**) TSB and (**B**) RPMI+ medium. dCas9 was expressed in the indicated CRISPRi strains using both the classical and vgp-CRISPRi systems to observe the impact of dCas9 on the growth of USA 300 LAC. WT does not contain the two plasmid-based CRISPRi systems. The growth curve was presented as means ± standard deviation from biological triplicate cultures.

Supplementary Information

Table S1 *E. coli* strains used in this study

Strains	Genotype and characteristics	Source or reference
IM08B	DH10B derivative, *Δdcm, Phelp-hsdMS, PN25-hsdS* (strain expressing the *S. aureus CC8* specific methylation genes)	Laboratory stock (33)
NEB® 10-beta	DH10B derivative, *Δ(ara-leu) 7697 araD139 fhuA ΔlacX74 galK16 galE15 e14- ϕ80dlacZΔM15 recA1 relA1 endA1 nupG rpsL (Str^R) rph spoT1 Δ(mrr-hsdRMS-mcrBC)*	New England Biolabs
Fluorescent reporter strains		
MR 1	IM08B carrying pCM29-P*sarA* P1-GFP, amp^R	This study
MR 2	IM08B carrying pCM29-P*atl*-GFP, amp^R	This study
MR 3	IM08B carrying pCM29-P*fnbA*-GFP, amp^R	This study
MR 4	IM08B carrying pCM29-P*coa* -GFP, amp^R	This study
MR 5	NEB® 10-beta carrying pCM29-P*sarA* P1-GFP, amp^R	This study
MR 6	NEB® 10-beta carrying pCM29-P*atl*-GFP, amp^R	This study
MR 7	NEB® 10-beta carrying pCM29-P*fnbA*-GFP, amp^R	This study
MR 8	NEB® 10-beta carrying pCM29-P*coa* -GFP, amp^R	This study

amp^R, ampicillin resistance

Supplementary Information

Table S2 Primers used in this study

Name	Sequence 5`-> 3`
RM 1_ P *atl* -KpnI F	CGG**GGTACC**TCTATTTATTACTCCTAAC
RM 2_ P *atl* -PstI R	AAAA**CTGCAG**GTTGTATCTATTTTAG
RM 3_P *fnbA* -KpnI F	CGG**GGTACC**TATAATATCTCCCTTTAAATG
RM 4_ P *fnbA* -PstI R	AAAA**CTGCAG**CTAAATATTAAGTAAACGTG
RM 7_ P *coa* -PstI F	AAAA**CTGCAG**CTTTGTTACTCCTTTG
RM 8_ P *coa* -KpnI R	CGG**GGTACC**AATTTTTTAATTCCTCC
RM 11_ P atl -SalI F	ACGC**GTCGAC**TCTATTTATTACTC
RM 12_ P *atl* -AvrII R	GCC**CCTAGG**GTTGTATCTATTTTAG
RM 13_ P *fnbA* -SalI F	ACGC**GTCGAC**TATAATATCTCCCTTTAA
RM 14_ P *fnbA* -AvrII R	GCC**CCTAGG**CTAAATATTAAGTAAACGTG
RM 15_ P *coa* -AvrII F	GCC**CCTAGG**GCTTTGTTACTCCTTTG
RM 16_ P *coa* -SalI R	ACGC**GTCGAC**AATTTTTTAATTCCTCC
RM 17_*gfp* check R	GTTACAAACTCAAGAAGGACC
RM 18_P*sarA* P1 check F	GACTCTAGAGGATCC
MK 44_*dcas9* check R	TGTCCGTTTGAGACGAGTC
MK25_sgRNA check R	AAATCTCGAAAATAATAGAGGGA
MK 26_sgRNA check F	GGATAACCGTATTACCGCCT
RM 19_ *rrsA* F	ACGGTCTTGCTGTCACTTATA
RM 20_ *rrsA* R	TACACATATGTTCTTCCCTAATAA
RM 21_*coa* F	GACCGCAATTTAACAAAACACC
RM 22_ *coa* R	AGCTCCGTATGATACTTGACC

F, Forward; R, Reverse

Supplementary Information

Table S3 Sequences of sgRNA base pairing regions

**Target**	**Sequence 5`-> 3`**
pbp1	GAACGAGGAAAGATATATGA
coa	TGGAGATACAGACAATCCAC
luc (no-target control, NTC)	CGGCGCCATTCTATCCTCTA
